# “Controlled by the game”– interviews on why regaining control over gaming is difficult for patients with gaming disorder

**DOI:** 10.1186/s40359-026-05258-y

**Published:** 2026-08-19

**Authors:** Annika Hofstedt, Kerstin Ulin, Anna Söderpalm Gordh

**Affiliations:** 1 Department of Addiction Disorders, Clinic for Gambling and Gaming Disorders, Sahlgrenska University Hospital, Gothenburg, Region Västra Götaland Sweden; 2 Institution for Neuroscience and Physiology, Section for Psychiatry and Neurochemistry, Sahlgrenska Academy, University of Gothenburg, Gothenburg, Sweden; 3 Institute of Health and Care Sciences, Sahlgrenska Academy, University of Gothenburg, Gothenburg, Sweden; 4 Sahlgrenska University Hospital, Gothenburg, Region Västra Götaland, Sweden

**Keywords:** Gaming disorder, Qualitative study, Clinical population, Treatment

## Abstract

**Background:**

Research on gaming disorder has grown substantially over the past two decades, but few studies have used qualitative methods. The focus on quantitative studies risks overlooking nuances of gaming disorder. For treatment purposes it is important to have knowledge about the full range of factors maintaining gaming disorder. The aim of this study was to explore how individuals with gaming disorder experience their gaming, with a particular focus on factors that make it difficult to control the amount of time spent gaming.

**Methods:**

Twelve patients diagnosed with gaming disorder were recruited at an outpatient clinic treating gaming disorder in Gothenburg, Sweden. Semi-structured interviews were conducted to examine factors that hindered them from reducing their gaming. The interviews were transcribed verbatim and analysed using thematic analysis.

**Results:**

Thirteen subthemes were identified, forming five main themes: *Being committed*, *Enjoying the game*, *Filling the void*, *Climbing an eternity ladder* and *Being controlled by the game*. The themes represent a wide range of reasons why it is difficult to control the amount of gaming, including aspects of the games, individual characteristics of the gamers and their life circumstances outside the games.

**Conclusions:**

The results highlight that gaming disorder is multifaceted and maintained by a combination of reduced self-control, structural game characteristics and the fact that gaming satisfies many basic human needs. For clinical purposes, it is important to address game-specific challenges, individual vulnerabilities and factors related to everyday life outside gaming to deliver effective treatments.

**Supplementary Information:**

The online version contains supplementary material available at https://doi.org/10.1186/s40359-026-05258-y.

## Highlights

In this qualitative study, patients describe multiple interacting factors that make gaming difficult to control.

Gaming disorder is maintained by a combination of reduced self-control, game design features, and the fulfilment of many basic human needs through gaming.

Treatment should address game-specific challenges, individual vulnerabilities and everyday life factors beyond gaming.

**Supplementary Information:**

The online version contains supplementary material available at https://doi.org/10.1186/s40359-026-05258-y.

## Background

Gaming is a common leisure activity, estimated to engage 40% of the global population [1]. In parallel with the increasing popularity of gaming, concerns have grown about its negative consequences, which is reflected by the inclusion of gaming disorder (GD) as a diagnosis in ICD-11 [2]. Gaming disorder implies impaired control over gaming, prioritising gaming over other activities and the occurrence of associated negative consequences. Prevalence is estimated at 1.39–1.96% [3, 4], which is comparable to gambling disorder (1.29–1.41%) but about half as common as alcohol dependence [5–7]. Research about GD has increased significantly over the last two decades. However, most studies remain quantitative and involve non-clinical populations [3, 8–11]. Moreover, research is scarce about factors maintaining GD, although such information is valuable for treatment purposes. Since there is a lack of qualitative research, this study aims to gain a deeper understanding of how patients with GD experience gaming and how GD is maintained.

Qualitative studies including highly engaged gamers have shown that gaming is experienced as a deeply meaningful activity and that their identity as a “gamer” can form an important aspect of their self-concept [12, 13]. Several studies have also examined motives for gaming in non-problematic gamers, identifying a broad variety of reasons, including social interaction, relaxation, and competition [13–15]. Using gaming for emotional escape has repeatedly been identified as the motive most strongly associated with GD, followed by achievement motives [16, 17]. The use of gaming as a coping mechanism aligns with research showing correlations between GD and other psychiatric conditions, such as anxiety, depression, ADHD and autism [18–24].

The development of GD is influenced by multiple factors. Qualitative work has shown that problematic gaming can emerge from unmet developmental needs and be intensified by poor parental supervision, limited social support, academic difficulties, and low self-efficacy [25]. A meta-analysis of modifiable risk factors identified individual factors as most influential, with gaming time and mood disturbance most strongly associated with GD, while self-control and conscientiousness were identified as the strongest protective factors [26]. Other identified risk factors include younger age and male gender [4]. Factors that contribute to GD can also be classified into game-related features (e.g., in-game rewards), personality traits and comorbidities, as well as environmental aspects (e.g., family functioning) [19].

Although several factors have been identified as contributing to the development of GD, the processes that maintain the condition may partly differ from those involved in its onset. Risk factors for GD include individual vulnerabilities, environmental factors and game-related features [19, 26, 27]. However, addiction models such as the I-PACE model suggest that as gaming develops into an addiction it gradually shifts from being a consciously controlled behavior to becoming seemingly automatic and less controlled [27, 28]. Clinically, understanding these maintaining processes may be especially informative for designing effective treatments.

Qualitative research on factors maintaining GD is limited. Existing studies suggest that time spent gaming may be influenced by prevalent exposure to games, idleness and stress, the use of gaming as a coping method or a social activity, in-game rewards and the sense of accomplishment that can be achieved through gaming [29, 30]. These findings suggest that the persistence of GD may be driven by a complex interplay of different factors underscoring the need for more in-depth qualitative research to inform tailored interventions. To date, no qualitative study has exclusively investigated what patients perceive as factors maintaining GD. Therefore, this study explores what factors patients with GD describe as making it difficult to regain control over their gaming.

## Methods

### Researcher characteristics and reflexivity

The interviews were conducted by the first author (AH), a clinical psychologist and PhD-student. The second author (KU) is an Associate Professor and trained nurse with extensive training and experience in conducting qualitative research but has no former professional experience of working with behavioral addictions. The third author (AG) is a Professor in experimental psychiatry with extensive knowledge and experience of conducting research relating to addiction. All researchers were women. To mitigate biases throughout the analytical process, the research team continuously discussed how their different backgrounds could influence the interpretation of the data. Our different backgrounds with a combination of having clinical experience of gaming disorder versus having experience from a separate research field, contributed with valuable complimentary perspectives, and helped our interpretations to be grounded in the interview data rather than preconceptions based on clinical and research based experiences of gaming disorder.

### Theoretical framework

This exploratory study used semi-structured interviews with patients diagnosed with GD. The data were analysed using reflexive thematic analysis, a qualitative method for identifying and interpreting patterns within data [31, 32]. The analysis was informed by a realist (essentialist) epistemological standpoint.

### Participant selection and setting

The study was conducted at the Department of Addiction Disorders, Sahlgrenska University hospital in Gothenburg, Sweden, at the Clinic for Gambling and Gaming Disorders (*Mottagning för Spelberoende och Skärmhälsa)*, an out-patient clinic specialised in the treatment of gaming disorder and gambling disorder. The clinic accepts patients with GD from the year they turn 16, with no upper age limit. The treatment provided is based on cognitive-behavioral therapy (CBT). Patients that fulfilled diagnostic criteria at intake assessment were asked to participate in the study. The diagnostic assessment was made by clinicians using a semi-structured diagnostic interview covering the criteria for Internet Gaming Disorder in the DSM-5 [33]. Patients were approached about the study via a clinician either during the assessment interview or during a treatment session, conducted face-to-face or as a video conference. Patients were also explicitly informed that their decision to participate or decline participation would not affect their treatment plan at the clinic. Fourteen patients agreed to participate at this initial question, but two were not included because they did not return the consent form. Thus, the final sample comprised 12 participants.

### Data collection

Individual semi-structured interviews were conducted by the first author (AH) between April 2021 and January 2022, with patients enrolled continuously using convenience sampling, however strategic in terms of only including patients with GD diagnosis. The interviewer had no therapeutic relation with the participants. All participants were informed about the goal of the research team to use the results from the study to increase the knowledge about individuals seeking treatment for GD and to inform the development of treatments for GD. The interviews were conducted individually via video conferencing, guided by a protocol developed for this study, aiming to explore the patients’ experiences of barriers to gaining control over their gaming. Questions explored participants’ current experience of the most positive aspects of gaming and factors within or outside games that were an obstacle to reducing play. Sociodemographic data and preferred gaming genres were collected. The interview guide was reviewed by individuals with extensive experience of videogaming assessing the relevance of the questions. In addition, each interview ended with questions on whether the interview guide covered the most important aspects in relation to the research question. As the participants stated that the interview had covered their experience of gaming in a comprehensive way, and none of the participants had any suggestions about additional questions, no modifications were made during the data collection process. Interviews lasted 21–79 min, were audio-recorded, and transcribed verbatim. During and directly after each interview, field notes were taken about initial reflections. The study did not include repeat interviews or member checking of transcripts or findings. None of the three researchers had a therapeutic relationship with the participants, and the participants were informed that their ongoing care would not be affected by their decision to participate or decline participation in the study.

### Participants

Twelve participants were recruited to the study. The mean age was 29.3 years (range 17–51). The three most preferred gaming genre was Multiplayer Online Battle Arena (MOBA) for example League of Legends, followed by mobile games such as Pokémon GO and strategy games for example Hearts of Iron. Half of the participants were interviewed before initiating the CBT-based treatment offered at the clinic, while the other half were interviewed after treatment (see Table 1).

**Table 1:** Participant demographics

Identifier	Sex (M/F)	Age (years)	Interview duration (min)	Treatment process (before/after CBT)	Preferred game genres
P1	M	27	70	Before	MOBA, Rougelike, Mobile games
P2	M	34	73	After	MOBA
P3	M	23	75	After	MMORPG
P4	M	36	73	After	MMORPG, Strategy games
P5	M	25	64	After	Strategy games
P6	M	29	79	After	MOBA
P7	M	17	21	Before	Strategy games, Action games
P8	M	51	76	After	Mobile games, MOBA
P9	M	19	22	Before	First-person shooter
P10	F	21	45	Before	Role-playing games, Mobile games
P11	M	38	40	Before	Mobile games
P12	M	26	42	Before	MOBA

*CBT* Cognitive- behavioral therapy, *MOBA* Multiplayer online battle arena, *MMORPG* Massively Multiplayer Online Role-Playing Game

### Data analysis

During analysis our themes were developed from the data, making the analysis primarily inductive. In the interpretation stage and formation of main themes, the themes were related to existing theoretical models of addictive behaviors.

The interviews were transcribed by the first author (AH) and two research assistants. The interviews were conducted in Swedish, and the analysis was made using the original Swedish data. The illustrative quotes reported in this article were translated into English by the authors and then validated by a bilingual professional translator. Data familiarisation was achieved by repeated reading of the transcripts, in addition to AH having conducted the interviews and transcribed some of them. Initial codes were generated from the transcripts by AH, using the qualitative data management software NVivo (version 14.23.0). 

To generate the codes, transcripts were analysed in relation to the research question. Codes were assigned to those sections of the interviews that covered either (a) gaming related aspects that were experienced as positive and rewarding and hence pulling towards gaming or (b) other factors in or outside the games that made it difficult to control the amount of time spent gaming. The second (KU) and third (ASG) authors independently read two transcripts to validate the coding and to ensure that no information relevant to the research question had been omitted. In the analysis of the twelfth interview, no substantially new patterns relevant to the research aim were identified. Due to the elaborate accounts provided by the participants, we judged that the data was rich enough to answer the research question. At this point, the dataset was considered to provide sufficient depth and breadth to support a coherent and well-developed thematic analysis, and no further interviews were conducted. 

Thereafter, AH organised codes with similar content into preliminary subthemes with provisional names, which were subsequently refined by all three authors. The analysis continued until no new themes were identified and consensus about the subthemes was reached among all the authors. The subthemes were then reviewed by all authors to identify overarching main themes. During the analytic process, particular attention was paid to distinguishing between themes that were conceptually related. For example, themes capturing structural features of gaming were analytically separated from those reflecting participants’ internalized experiences, to preserve distinctions between external conditions and lived consequences. Finally, the themes and subthemes were evaluated in relation to the broader context of GD, forming the basis for the discussion section.

## Results

The participants described a wide range of reasons why they found it difficult to control their gaming. Thirteen subthemes were identified during the analysis, forming five main themes: *Enjoying the game*,* Climbing an eternity ladder*,* Being committed*, *Controlled by the game*, and *Filling the void*. The main themes, subthemes and examples of quotes from each theme are shown in Table 2.

**Table 2:**
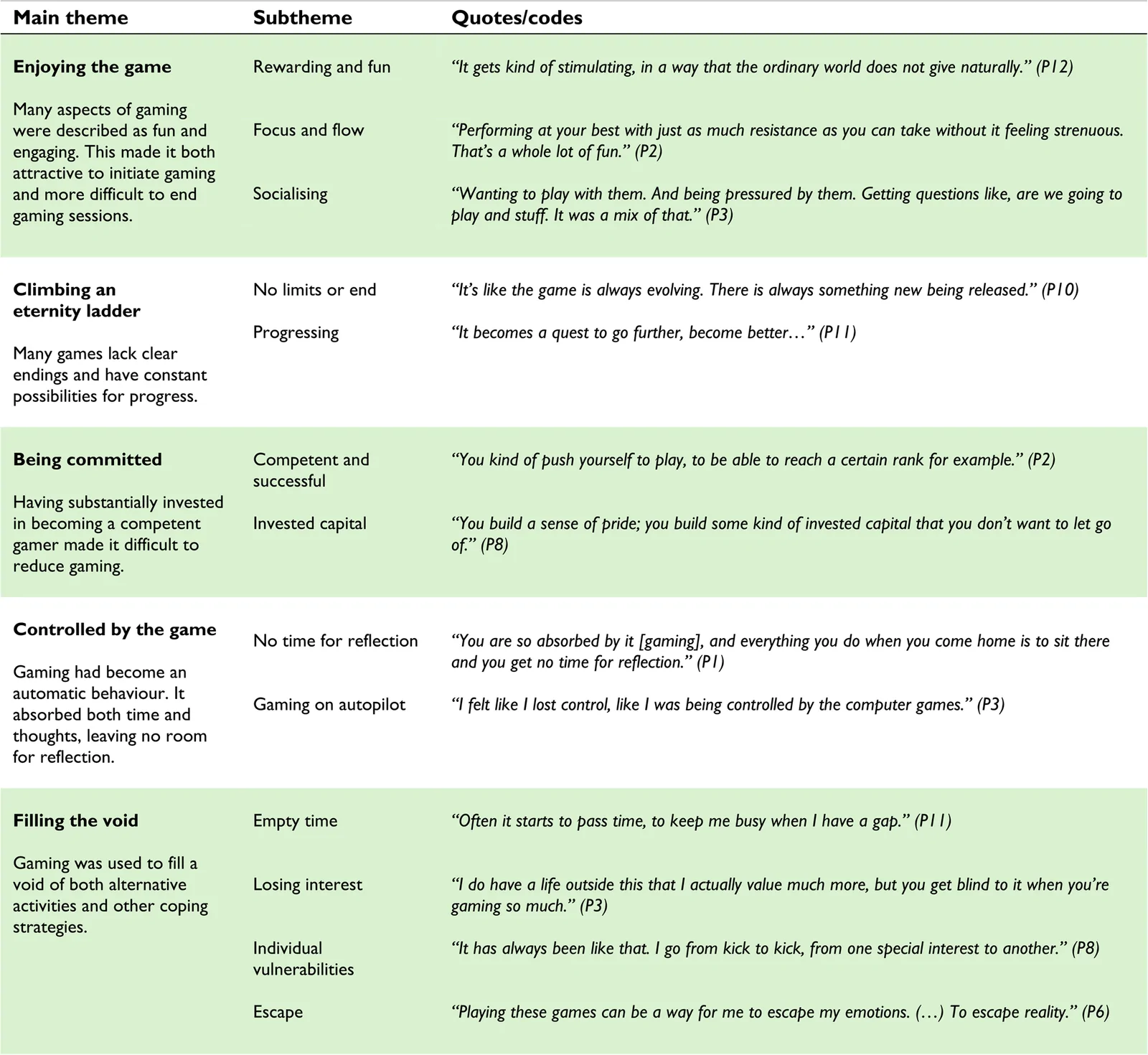
Themes, subthemes and corresponding quotes describing why it is difficult to control gaming

The table shows main themes (written in bold, followed by a short description), subthemes and one illustrative quote (in italics) per subtheme

### Enjoying the game

The participants described many different aspects of gaming as fun and engaging; these experiences both attracted them to gaming and made it more difficult to end gaming sessions.

#### Rewarding and fun

Many noted that the frequently distributed in-game rewards made it tempting to play longer. Some of the rewards were described as gambling-like, where luck decided if the rewards were received quickly or after a lot of time and effort. Rewards could take many different forms, from simple stimuli like sound effects to receiving highly valued in-game items.

*“Stimulation and rewards all the time. Rewards work very well on me. It’s everything from a happy little ‘ping’ that means that you’ve done something good*,* to getting access to the other part of the world*,* on the world map.” (P8)*.

The rewarding properties of gaming were described as much more powerful than experiences outside gaming, making gaming a preferred activity.

*“It gets kind of stimulating*,* in a way that the ordinary world does not give naturally.” (P12)*.

Other enjoyable aspects included the possibility of entering a totally different world and experiencing appealing visual aspects of the games. Participants noted that while not all gaming time was equally enjoyable, the most engaging moments made quitting especially hard.

*“There are boring and there are enjoyable parts of gaming. And if you’re in one of those fun parts of the game then it is fun to continue. Then you don’t want to quit.” (P5)*.

#### Focus and flow

The participants appreciated the experience of total focus when gaming; it felt stimulating and captivating. The level of focus when gaming was described as much higher than they experienced in life outside gaming.

*“Like my focus now*,* today*,* it cannot be directly compared at all with the level of focus I had when gaming. When I’m gaming*,* my level of focus can be 100% but now*,* when I do things that are so-so*,* kind of fun*,* then maybe my level of focus is 70%.” (P6)*.

Gaming also offered an arena to achieve a flow-like state when gaming on a level perfectly suited to your skills.

*“Performing at your best with just as much resistance as you can cope with without it feeling like hard work. That’s a whole lot of fun.” (P2)*.

#### Socialising

Having contact with friends or getting new friends was also described as a factor that attracted the participants to gaming.

*“I was looking for long-term connections with the other players*,* and I got that because I spent so much time playing which meant I could be part of the higher-ranked groups.” (P10)*.

Some described having few other alternative ways to socialise, as most of their friends used gaming and the internet as their main social arena.

*“Most people sit by the computer when they are not at work*,* in school or other things. If you’re going to hang out somewhere*,* the Internet is the place. And there must be a good reason why you should do something else*,* in real life. People want it to be worth their while nowadays.” (P12)*.

Being able to play together, for example, in team-based games that require a lot of co-operation, was described as positive. However, it could also involve feeling pressured to join the game.

*“Wanting to play with them. And being pressured by them. Getting questions like*,* are we going to play and stuff. It was a mix of that.” (P3)*.

Participants described the social aspects as important reasons for wanting to continue their engagement in gaming, even after treatment.

*“The most important thing for me was really the social side*,* which I’ve chosen to try to keep.” (P4)*.

### Climbing an eternity ladder

Structural characteristics of the games were mentioned as factors driving engagement. The combination of games with no clear endings yet constant possibilities for progress was described as captivating. For some participants, the lack of limits within the games was reinforced by a lack of limitations on gaming in the social context outside gaming.

#### No limits or end

The games were described as limitless in many ways; there is always something new to discover and it is almost impossible to build a perfect character.

*“And what happens when I get my reward*,* reach that goal and look ahead? Oh*,* there is a new challenge and a new reward to strive for. (...) Like some kind of eternity ladder.” (P4)*.

In addition, new updates or time-limited challenges are regularly released, driving engagement.

*”It’s like the game is always evolving. There is always something new being released.” (P10).*

Some described having no help from relatives or friends in setting limits – either few people had insight into their gaming, or none objected to the amount of time they spent playing.

#### Progressing

The constant possibilities to progress and develop one’s game character were described as significant reasons for the difficulty in limiting gaming time.

*“I think most games are like this—that you become stronger*,* but you don’t really know exactly how it happens. I think that’s the secret recipe that keeps me hooked.”(P1)*.

This could evolve into an urge or perceived pressure to achieve in-game goals.

*“It becomes a quest to go further*,* become better…” (P11)*.

Some described how the path forward in the game felt much clearer than in real life, and this made the progression in the game even more captivating.

*“In the rest of your life*,* maybe it isn’t always so very clear how to move forward and develop. You just have to take a step into the unknown with trial and error and see if that works in relation to work*,* friends*,* activities and the rest of life. (…) while in the game you have a well-lit highway that is flat*,* and you see rewards ahead of you at regular intervals.” (P4)*.

### Being committed

Many participants described having made substantial investment and effort to become a competent gamer, and this made it difficult to reduce their gaming. Although *Climbing an Eternity Ladder* is closely related to *Being Committed*, these main themes capture different aspects of the experience: the former reflects structural features of gaming that sustain engagement, whereas the latter describes how these features are gradually internalized as commitment over time.

#### Competent and successful

The process of developing gaming competence was described as enjoyable and stimulating, but the pressure to succeed could also take the joy out of gaming.

“*Over time*,* it has become more important how skilled I am at the game*,* and less important how fun it is.”* (P12).

Some participants described gaming as a potential career plan and framed their gaming more as a work-related activity than a source of entertainment.

*“Because I was extremely ambitious in CS*,* you know. I really wanted to go pro and all that.”(P3)*.

Several participants further described themselves as being competitive and gaming therefore became an arena for performing and winning.

*“It’s more or less that I play because I’m a very competition*-*oriented person*,* I think*,* at least right now.” (P7)*.

In-game ranking systems were described as valuable for getting feedback about performance, but some also described a struggle to increase or retain their rank.

*“You play even though you’re not having fun*,* and though you don’t really feel like it. So you kind of push yourself to play*,* to be able to reach a certain rank*,* for example.” (P2)*.

Performing well in the games was described both as a way of impressing others and increasing self-confidence. For some, gaming provided an arena in which to feel competent, which was especially important when they were not feeling competent in other areas of life.

*“I didn’t feel successful at work. Sometimes I felt like a bad parent. I didn’t have the energy to renovate the house at the same time. And then I lost my job… So it was also like: here [in the game]*,* here is a place where I can be good. That kind of validation in a way.” (P11)*.

#### Invested capital

The participants described difficulties leaving the game when they had spent so much time, and in some cases, also money, developing their game characters. They expressed feelings of pride and emotional attachment to their game characters – bonds that in some cases had been built over many years.

*“With role-playing games*,* it’s very much about building your character—from level one and upwards—and then you develop a kind of attachment to that character*,* I think.” (P10)*.

These accumulated investments made it difficult to disengage from gaming.

*“You might have a gaming character*,* and it can be improved and all that. Then you build a sense of pride; you build some kind of invested capital that you don’t want to let go of.” (P8)*.

Some participants held positions of responsibility within the game, such as being a team leader or having the status as top-performing player on the server. These roles came with expectations from other players, for example, to assist in quests and other tasks, further reinforcing their sense of obligation to the gaming community.

*“It kind of becomes a… well*,* I’m not sure if you can call it a positive spiral*,* maybe it’s more of a negative one*,* but as you become more and more competent and skilled at the games because you put so much time into them*,* you also become more sought after by acquaintances and friends in the game. They want help or start asking questions.” (P4)*.

Being a gamer was described as a central part of their identities and had been so for a long time.

*“Somewhere deep down*,* there’s a little gamer in me who enjoys that nerdy stuff; painting plastic miniatures and playing board games. I grew up with computer and console games*,* that whole culture.” (P2)*.

Some noted that their time spent gaming had fundamentally changed their view and interpretation of the world, making it difficult to feel fully understood by people outside the gaming community.

*“It kind of feels like it has evolved into an “us” and “them”– us*,* the ones who’ve been gaming a lot*,* and the rest of the world who haven’t been gaming.” (P1)*.

### Controlled by the game

Many described how gaming gradually had become more automatic, and this shift contributed to a sense of losing control over the time spent playing. Gaming absorbed both their time and thoughts, leaving no room for reflection.

#### No time for reflection

Participants described becoming so absorbed in gaming that it was difficult to gain the time and distance needed to reflect on their situation.

*You are so absorbed by it [gaming]*,* and all you do when you come home is go sit there and you get no time for reflection. (P1)*

Some described experiencing a cognitive overload due to intensive gaming that negatively affected their capacity for reflection.

*“It’s full focus all the time*,* so I think that kind of thing can maybe create burnout*,* sort of.” (P12)*.

The threshold for starting to play was lowered by rationalising thoughts, for example, by telling themselves they would only play for a short while or that they might as well spend time gaming when not being productive in other responsibilities anyway.

*“It’s even worse when you’re working from home*,* and you catch yourself having spent half an hour or an hour just thinking about the game and not getting anything done at work. And then you end up thinking*,* well*,* I might as well actually sit down and play instead*,* because I’m not getting anything done at work anyway.” (P4)*.

#### Gaming on autopilot

Gaming was described as an activity that over time had become a habit.

*“I don’t really feel that I have a genuine need to game anymore; instead*,* it’s more become a routine; something my brain expects. Kind of like how dinner is served around five or six in the afternoon.” (P1)*.

Participants reported having lost control over gaming to the extent that it no longer felt like a conscious decision to engage in it.

*“I felt like I lost control*,* like I was being controlled by the computer games.” (P3)*.

The habit or compulsion resulted in gaming, even when they were not in the mood for it, resulting in feeling powerless in relation to their gaming.

*“All the time drawn to something that you really didn’t want. Because you were sitting there at the computer*,* and lots of times you gamed without even feeling like it.” (P3)*.

### Filling the void

Gaming was used to fill empty time, which otherwise could be experienced as distressing or difficult to endure. Participants also used gaming to escape from other types of unpleasant thoughts or feelings. This use of gaming as a coping mechanism was further driven by general problems with self-control, and a gradually declining interest in other activities. Over time, gaming had come to dominate participants’ daily routines, even at the expense of other highly valued activities. Thus, gaming was used to fill a void of both other activities and other coping strategies.

#### Empty time

Most participants cited a lack of alternative activities as a key reason for difficulty in reducing their gaming time.

*“…to try to find something that can fill it…*,* the void…*,* because I had like nothing else to do.” (P5)*.

The lack of other activities resulted from having given up previous hobbies, unemployment or having work or studies that were not fully demanding.

*“But when you don’t have a job*,* it’s like. you have to fill those hours somehow.” (P12)*.

They also mentioned shorter periods of unoccupied time, which were often filled with gaming.

*“Often it starts to pass time*,* to keep me busy when I have a gap*,* for example*,* when I’m on the train or sitting on the toilet*,* kind of… It often begins like that.” (P11)*.

Some participants also described having few or no friends, limiting their opportunities for social engagement and leaving them with more spare time.

*“You feel a bit lonely and have a lot of time to kill.” (P11)*.

#### Losing interest

When gaming excessively, other aspects of life gradually lost priority, feeling less important and more monotonous.

*“And then the game becomes a task you really care about in itself*,* and then I don’t feel the same passion for other things anymore. They feel more boring.” (P8)*.

Even activities or areas of life that the participants highly valued were overshadowed by gaming.

*“I do have a life outside this that I actually value much more*,* but you get blind to it when you’re gaming so much.” (P3)*.

#### Individual vulnerabilities

Participants described vulnerabilities that made it difficult for them to regulate the amount of time spent gaming. Most reported difficulties with self-control not only in relation to gaming but also across other areas of life.

*"I don’t know if it has to do with me being kind of a dopamine addict*,* that I need to have something to do to feel okay*,* or if it’s just how my brain is wired. I have always been a restless person*,* like in school and all that." (P12)*

They described a tendency to become deeply absorbed in different activities, often engaging in them excessively.

*“It has always been like that. I go from kick to kick*,* from one special interest to another.” (P8)*.

Sometimes, individual vulnerabilities became intertwined with gaming, amplifying each other.

*“My health got worse because I worried for so long during the days*,* and then I had trouble sleeping at night. And because I had trouble sleeping*,* I was gaming at the same time*,* which led to sleep problems… and then*,* well… everything sort of came in a wave.” (P10)*.

#### Escape

Escaping reality and coping with unpleasant thoughts or feelings through gaming was a recurring theme.

*“Playing these games can be a way for me to escape my emotions. (…) To escape reality.” (P6)*.

The unpleasant thoughts and feelings could, for example, be related to academic difficulties or feeling stressed or depressed for various reasons. Gaming was used as an escape from many different hardships in life.

*“Of course*,* when you feel a bit down*,* it’s easier to use gaming as an escape*,* when things are rough in a relationship*,* when you’re a bit depressed for one reason or another*,* feeling low*,* or maybe when the weather is boring and there isn’t much else to do.” (P4)*.

Specifically, many described restlessness and monotony as almost painful conditions that they often alleviated with gaming.

*“I’m sitting at work*,* being understimulated*,* and I get these bad feelings*,* like “I can’t have it like this forever*,* it doesn’t work*,* it’s like torture.” (P12)*.

Some described the possibility of using gaming for escape as potentially positive, if used for shorter periods to relax or unwind. However, using gaming this way could create a negative spiral, providing temporary relief but contributing to an increase in negative emotions over time.

*“As soon as something went bad I only turned to the games instead… I needed it in some way to feel better*,* even though feeling better was only temporary. As soon as I stopped playing I felt bad again*,* and then I needed to play again to feel better. It turns into a negative spiral…” (P10)*.

## Discussion

**Fig. 1:**
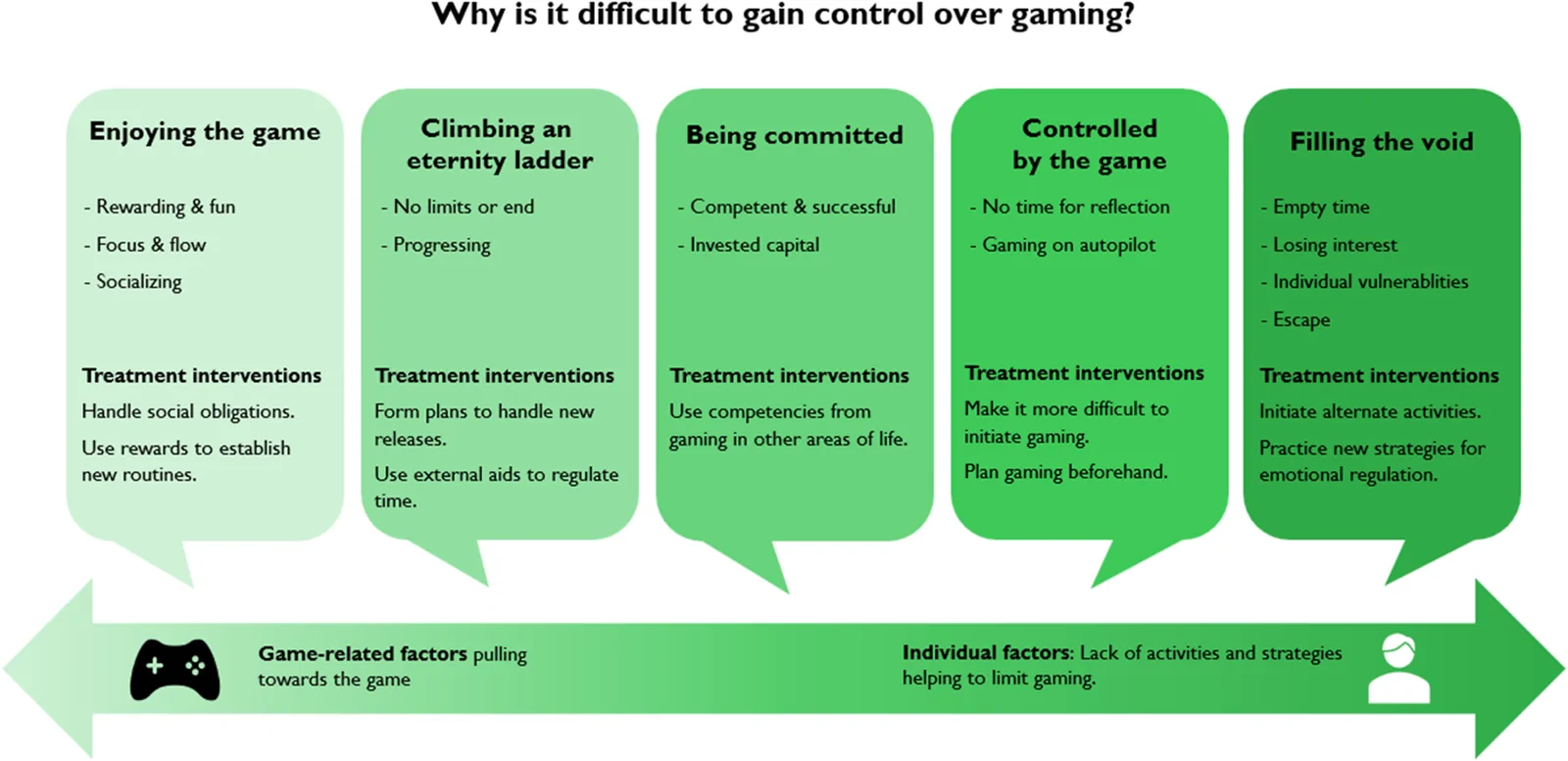
Main themes, subthemes and corresponding examples of possible interventions in treatment Note: The main themes are placed in an approximate order, from those mostly concerning game-related factors pulling towards gaming to those mostly relating to individual factors that make it difficult to abstain from gaming

This study highlights the multifaceted nature of GD. The themes represent a wide range of reasons why it is difficult to control gaming, including game design, individual vulnerabilities, life circumstances and how gaming corresponds to basic human needs. These reasons and examples of matching treatment interventions are illustrated in Fig. 1, with themes placed in an approximate order ranging from game-related factors pulling towards gaming to individual factors that impede control over gaming.

Many aspects contributed to the participants’ *enjoyment of the games*, ranging from getting rewards and socialising to experiencing deep focus. During the development of an addiction, compensatory effects often come to dominate over gratifying effects [27]. However, the breadth of positive effects mentioned by the participants highlights the importance of acknowledging the simultaneous existence of positive, negative and compensatory effects, which is in line with recent clarifications of the I-PACE model for behavioral addictions [28]. This updated interpretation of the I-PACE model suggests that gratification may remain stable throughout the addiction process, as illustrated by the many rewarding experiences reported by our participants, accompanied by gradual increase of compensatory effects. The participants stated that in-game rewards were often distributed at random intervals. This adds addictive potential to the games [19, 34], as intermittent rewards are known to be especially addictive [35]. 

Additionally, social aspects were mentioned as important positive factors. Social interaction is an integral part of many different games [13]. However, gaming for social reasons can also have negative consequences, as research shows that social motives for gaming are associated with GD, particularly when in-game relationships create a sense of “social obligation” [16, 17]. At our clinic, the enjoyment of gaming is reflected in most patients formulating treatment goals aimed at achieving controlled gaming, i.e., continued gaming that causes fewer negative effects. The participants in our study highlighted social motives as the key reason for continuing gaming. Clinically, analysing these motives may help enable social gaming while mitigating the risk of excessive play. Treatments can also capitalise on the effectiveness of rewards by encouraging patients to reward themselves after taking steps toward their treatment goals.

Gaming was further described as *climbing an eternity ladder*, meaning that many games lack a clear endpoint and instead promote endless progression, making self-regulation of playtime more difficult. This theme highlights the relationship between game design and difficulties in limiting gaming. Others have also identified the permanent nature of the game world and other structural characteristics of the games as important for maintaining gaming over time [34, 36–38]. The use of new releases is also part of the gaming industry’s strategy to strengthen commitment over time [1]. Clinically, it could be useful to address these game-specific challenges in treatment, for example, by forming plans on how to handle new releases or using external reminders, like timers, to regulate gaming time.

The main theme *being committed* reflects the great effort that the participants have invested into gaming, making it an important part of their identity and a source of pride. This theme specifically captures the commitment that results from extensive gaming and interaction with structural characteristics of the games over time. From a psychological perspective, these experiences reflect the upper tiers of Maslow’s hierarchy of needs: the need for self-esteem and self-actualization [39]. As in other studies [12, 13], the participants in our study described gaming as intertwined with a sense of belonging to a subculture and that this influenced the gamers’ identities. Striving to be successful in the games involved considerable effort and competitiveness. Notably, competitiveness is one of the motives most strongly associated with GD symptoms [16, 17]. The considerable time and effort spent on gaming also made it harder to stop. One participant described this as having *“invested capital that you don’t want to let go of”.* This aligns with findings from cognitive psychology regarding the well-documented cognitive bias “the sunk cost fallacy” [40], which highlights that individuals are more likely to continue efforts after prior investment has been made. All in all, clinicians should acknowledge the meaningful aspects of gaming and incorporate them into treatment. This can be achieved, for example, by identifying in-game competencies and exploring how these skills can be used in other areas of life.

Descriptions of being *controlled by the game* and playing on autopilot closely mirror the diagnostic criteria regarding impaired control over gaming. Impaired control is considered a core diagnostic criterion of GD [41, 42], i.e., a criterion that effectively separates individuals with significant problems associated with gaming from non-problematic gamers. Habitual gaming is an important aspect to address in treatment. Helpful interventions in treatment could include creating external limits or obstacles regarding gaming. Some practical examples are to delete shortcuts to games or to place the keyboard away from the computer so that initiating gaming becomes a bit more complicated, thereby creating time and space for more deliberate decisions about gaming. Interventions could also include making plans about when gaming should take place. As the desired outcome for most patients is to continue gaming in a controlled manner, useful preventative measures make it more difficult but not impossible to engage in gaming.

Finally, *gaming to fill the void* captures descriptions of using gaming as a coping strategy and to fill empty time. The use of gaming as a (maladaptive) coping mechanism aligns with previous research showing that coping is the motivation most strongly associated with GD [16, 17]. Some participants described gaming as a potentially positive and unproblematic form of escape when used for shorter periods. However, they also emphasized an inherent risk of entering a negative spiral if gaming becomes the sole means of relaxing or unwinding. 

Relatedly, the importance of distinguishing between adaptive and maladaptive forms of escapism has been highlighted in the literature [43]. In the C-DOG model developed by Giardina et al., [44], one proposed distinction concerns the extent to which gaming is used to compensate for and process problems in physical life versus to avoid the physical environment. Another possible distinction is to differentiate adaptive and maladaptive escapism based on functional outcomes; namely whether aversive experiences are alleviated and the gamer can readily disengage from gaming, or whether gaming persists [45]. 

The need to use gaming for coping and escape may stem from concurrent psychiatric problems, such as ADHD [20] or autism [22], which are common among individuals with GD. Few participants in our study explicitly mentioned other psychiatric comorbidities. However, they described problems with self-control and impulsivity, noting that these traits made them more susceptible to gaming. 

Additionally, most participants mentioned that gaming was affected by the presence of empty time, suggesting that this aspect could be especially important in treatment. Implementing alternative activities in line with individual goals or making plans for how to handle empty time could help address this. Since most patients want to continue gaming, plans could include an analysis of which parts of gaming are most valuable. Such considerations can then guide decisions about when to spend time gaming, rather than basing it solely on the presence of empty time. Further, practising alternate ways of identifying and handling difficult emotions could reduce the need to use gaming for coping.

Findings from this study both confirm and expand the conceptualization of GD proposed in dominant frameworks such as the I-PACE model [27, 28]. Participants’ descriptions of enjoying the game while simultaneously feeling controlled by it, as well as using gaming to escape or cope with individual vulnerabilities, fit well with the increasingly habitual and compensatory behavior described as characteristic of later stages of addiction in the I-PACE model. However, our findings also illustrate additional maintaining factors that may be particularly important when refining models to better capture processes specific to GD, namely game-design, basic human needs and life outside gaming. These factors have been identified in previous GD research (e.g., Király et al., [19]. Yet, they are not described in detail in the I-PACE model, that is designed to be valid for many different types of addictive behaviors [27]. We suggest that these additional factors may be important to incorporate explicitly in a GD-specific version of the model, to better understand the multitude of factors that contribute to maintain problematic gaming.

The importance of game-design and game mechanics, such as endless gameplay and continuous opportunities for progress, is illustrated in the main theme *climbing* *an eternity ladder*. Previous studies have also highlighted the relevance of such design elements in relation to the risk of developing GD [37, 38, 46, 47]. While related aspects may be indirectly addressed, these gaming-specific features do not appear to be explicitly or systematically emphasized in frameworks such as the I-PACE model. These game-design aspects could therefore be more explicitly integrated into conceptual models of GD, as they may be important, for example, for better informing approaches aimed at enabling controlled gaming. The potential of gaming to fulfill a wide range of basic human needs is described in the main theme *being* *committed* and has also been noted in previous research [12, 13]. Although this aspect is partly addressed in the I-PACE model under predisposing individual characteristics, it may warrant more detailed treatment in a model adapted specifically to gaming. 

Finally, our results highlight the importance of life outside gaming, particularly unstructured time and a lack of alternate activities. These environmental factors may be valuable to explicitly integrate into theoretical models of GD, not least given the extensive amount of time often devoted to gaming by individuals with GD [48, 49]. Consequently, reducing gaming without simultaneously increasing engagement in other activities may create large amounts of empty time, which may in turn increase the risk of relapse.

This study has both strengths and limitations. The sample’s demographics (age, gender, games played) resemble those in larger clinical samples [48, 50], suggesting the findings may be representative of patients with GD. Another strength is that data collection was conducted by a single interviewer (AH) with no therapeutic relation to the participants. Limitations include the single hospital setting, although the national intake of patients to the clinic supports broader applicability. Self-reported data may involve recall or social desirability biases, even though the interviews were designed to encourage participants to respond truthfully and reflectively. The sample was limited to treatment-seeking individuals diagnosed with GD, excluding those with subclinical gaming problems and individuals with GD in the general population, which could have provided a more nuanced picture. The modest sample size (12 participants) and skewed gender distribution (only one female) may limit perspectives. Including participants interviewed both before and after CBT was necessary due to low referral rates at the time of the study. Having to limit inclusion to treatment-naïve participants would have substantially reduced the sample. The two groups contributed complementary perspectives with pre-treatment interviews capturing descriptions of experiences, whereas post-treatment interviews allowed for more reflective accounts of pre-treatment gaming. Although retrospective reports of experiences of gaming may have been influenced by therapeutic concepts introduced during CBT, this was considered an acceptable trade-off to ensure sufficient clinical variation and richness of the data.

Future research could strive to include samples with a larger proportion of females and compare GD patients with leisure gamers to better understand differences between problematic and non-problematic gaming. It may also be useful to design a study specifically to compare descriptions of the gaming experience before and after treatment to capture processes that changes during treatment. Future studies could also investigate whether and how the importance of these maintaining factors differs among patients with GD. It has previously been suggested that there are substantial differences between individual patients with GD regarding symptoms and etiology, which could be important for personalized treatment planning [51], for example by selecting specific modules and interventions from a CBT manual based on individual needs [52].

## Conclusions

This study identified a wide range of factors that make it difficult for patients with GD to control gaming. The findings align with theoretical models [27, 53] describing how addictive behaviors evolve from being consciously regulated to becoming more automatic and less controlled. However, the results also point to the importance of the games’ structural characteristics, gaming as an arena for competence and success, and the social environments of gamers outside gaming. Overall, this highlights the need for treatments to address a breadth of maintaining factors, from in-game features and individual characteristics to life circumstances beyond gaming.

## Supplementary Information

Supplementary Material 1.

Supplementary Material 2

## Data Availability

The datasets used and analysed during the current study are available from the corresponding author on reasonable request.

## Abbreviations

ADHD: Attention Deficit Hyperactivity Disorder
CBT: Cognitive Behavioral Therapy
DSM-5: Diagnostic and Statistical Manual of Mental disorders, 5th revision
GD: Gaming Disorder
ICD-11: International Classification of Diseases, 11th revision
I-PACE: Interaction of Person-Affect-Cognition-Execution
MOBA: Multiplayer Online Battle Arena
MMORPG: Massively Multiplayer Online Role-Playing Game
