# Supplementary material for: “Controlled by the game”– interviews on why regaining control over gaming is difficult for patients with gaming disorder
Source: BMC Psychol. 2026 Aug 19;14:1506. doi: 10.1186/s40359-026-05258-y (PMC13681908; doi:10.1186/s40359-026-05258-y)
Supplement: Supplementary file 2 [file 40359_2026_5258_MOESM1_ESM.docx]

Experiences of gaming disorder – interview guide

___________________________________________________________________________

**Instructions**

Main questions marked in bold. Examples of follow-up questions in italics. General follow-up questions to be used throughout the interview: Could you tell me more about that? Please tell me some more. Is there anything else you come to think of? How do you mean? Etc.

Introduction

**You’re in contact with the clinic because you’re seeking help for problems related to gaming. The purpose of this interview is to get a better picture of how gaming is experienced among those seeking treatment at the clinic.**

**To begin with, I’d like to get a picture of what your life is like right now.**

**What’s your life like right now? What does a typical day look like? And can you tell me about your gaming at the moment?**

**What kinds of games do you prefer? What makes you prefer those games?**

**What change do you hope to achieve? If that happens, how do you imagine your gaming and the rest of your life would be?**

- *What would be different compared to now, in your life and how you feel?*
- *What would you do more of or less of compared to now?*

Maintaining factors

**What are the most important positive and negative aspects of your gaming right now?**

- *How does it affect life outside gaming? Relationships? Occupation?*
- *What do you think others think of your gaming? Positive aspects and possible problems?*

**Have you tried to change your gaming habits?**

- *Why did you try to do that?*
- *Can you tell me about it?*
- *What did you do?*
- *How did it go? (what was the most difficult part of achieving the desired change?)*

**What is it like for you when you are not gaming, when it is not possible for some reason? How does it feel then?**

**Is there anything in the specific games you play that makes it especially difficult to reduce or abstain from gaming?**

**Is there anything in addition to the specific games you play that makes it especially difficult to reduce or abstain from gaming?**

- *In your life in general? How you feel, your life circumstances, something else?*
- *Are there any other difficulties that affect your gaming? Is gaming the only problem, or are there other challenges?*

**If you compare your own gaming to others you know who play video games and don’t seem to have major problems related to their gaming, do you notice any differences?**

Developing problematic gaming

**If we start from the beginning… How old were you when you started to play video games regularly? Tell me what your gaming was like then:**

- *What did you play? When? With whom?*
- *Can you describe the most important reasons why you started gaming, e.g. what functions did gaming have for you?*
- *How did you feel before gaming? While gaming? After gaming?*
- *How was your life during the period when you first started gaming?*

**Has your gaming changed since you started playing video games? How? Has the experience of gaming changed?**

- *How do you feel before gaming? While gaming? After gaming?*
- *Have there been any changes in what you experience as positive or negative aspects of gaming since you started gaming?*
- *Time allocated to gaming: has this changed? What would you say is the reason for changes in the time you spend gaming?*

**Can you tell me what it was like when you started to realise gaming wasn’t good for you – that gaming was a problem for you?**

- How did you notice it?
- Did you yourself notice or did other people seem to notice before you did? (Changes in how you gamed, school, family, friends, leisure activities or some other way? Did you stop doing things?)
- How did that lead to your seeking help?

The addiction terminology

**Excessive or problematic gaming is sometimes also called an addiction. How do you think the term addiction matches your own gaming?**

- What word do you yourself use to describe your gaming?
- Do you see any positive or negative sides to talking about it as an addiction?

To conclude

**Is there anything else you want to tell me that hasn’t been covered in the interview, and that you think would be good for me/us to know?**
